# A new potential mosquito-borne virus: detection of Human-derived Jingmenvirus in several-species of mosquitoes from Yaoundé, Cameroon

**DOI:** 10.21203/rs.3.rs-7347858/v1

**Published:** 2025-09-09

**Authors:** Lisandru Capai, Giovanni Begliomini, Basile Kamgang, Souand Mohamed Ali, Sarah Temmam, Thomas Bigot, Gisèle Liliane Machuetum, Christophe R. Keumeni, Francine Yousseu Sado, Christian Yogne Nsangou, Gael Dieudonné Essima, Landry Mounchili, Christian Meke, Vincent Kome, Rodrigue Poueme, Ahmadou Alkaissou, Richard Njouom, Paul Alain Tagnouokam-Ngoupo, Nolwenn M Dheilly

**Affiliations:** Institut Pasteur, Université Paris Cité; Institut Pasteur, Université Paris Cité; Centre for Research in Infectious Diseases; Institut Pasteur, Université Paris Cité; Institut Pasteur, Université Paris Cité; Institut Pasteur, Université Paris Cité; Centre Pasteur du Cameroun; Centre for Research in Infectious Diseases; Centre for Research in Infectious Diseases; Centre Pasteur du Cameroun; Centre Pasteur du Cameroun; Centre Pasteur du Cameroun; Laboratoire National Vétérinaire; Ministry of Livestock, Fisheries and Animal Industries; Laboratoire National Vétérinaire; Ministry of Livestock, Fisheries and Animal Industries; Centre Pasteur du Cameroun; Centre Pasteur du Cameroun; Institut Pasteur, Université Paris Cité

**Keywords:** Jingmenvirus, Mosquitoes, vector-borne diseases, NGS

## Abstract

**Background:**

Tick-borne Jingmenviruses are becoming an increasing arbovirus concern due to the rising number of reported infections in humans and animals, as well as their wide geographic distribution. The involvement of other hematophagous arthropods as vectors of Jingmenviruses is still unknown.

**Methods:**

Mosquitoes were sampled in two different biotopes in Cameroon (Yaoundé and Garoua) during the rainy and the dry seasons in 2022 and 2023. Metatranscriptomics Next Generation Sequencing was conducted using Illumina technology. Viral sequences detection revealed the presence of several contigs with high sequence identity to a human-derived Jingmenvirus (HdJV) previously discovered in plasma from an individual from Yaoundé, Cameroon. A draft viral genome was constituted for each Jingmenvirus-positive samples. Maximum likelihood phylogenetic reconstructions were used to position mosquito-associated viruses within the diversity of Jingmenviruses. Statistical analyses were conducted to estimate the prevalence of infected mosquitoes and the effect of different variables (region, season, year, mosquito species) on Jingmenvirus detection.

**Results:**

HdJV was identified during the dry and the rainy seasons in 4 species of mosquitoes: Aedes albopictus, Culex quinquefasciatus and Culex wansoni from Yaoundé, and Anopheles gambiae s.l. from Garoua. The overall prevalence of HdJV-infected mosquitoes was estimated to 0.90% [0.41–1.69]; and the unique variable significantly associated with HdJV detection was the sampling area: Yaoundé showed the highest prevalence (2.29% [0.95–4.68]) compared to Garoua (0.18% [0.01–0.79]). Mosquito-associated Jingmenviruses shared a high nucleotide identity (between 98.64–100% according to the segment) and clustered in the same clade in the phylogenetic analysis, that they belong to the same viral species circulating in different mosquito species. The viral genome shared between 96.4% and 98.9% nucleotide identity with a HdJV detected in the plasma of a patient suffering from febrile illness originating from the same area, suggesting the possible involvement of mosquitoes as vectors of arboviral Jingmenviruses in human infections.

**Conclusions:**

This finding provides new insights into the ecology and transmission dynamics of Jingmenviruses, highlighting mosquitoes as potential vectors, alongside ticks, in the zoonotic transmission of this virus group.

## Background

Jingmenviruses represent a growing concern amid the increasing number of reports of infection in humans and animals, their large geographic distribution and their broad host range (1). Jingmenviruses are a group of positive-strand RNA viruses - not yet classified by the International Committee for the Taxonomy of Viruses (ICTV) - that have a segmented RNA genome (1) consisted of four to five segments that encode for up to seven structural proteins and two non-structural proteins, the latter sharing significant similarities with the non-structural proteins (NS2B/NS3 and NS5) of flaviviruses (2–5). Jingmenviruses are classified into two phylogenetic clades, typically referred as the “tick -associated” clade (that contains many vertebrate-associated Jingmenviruses, including the human pathogen Alongshan virus (6) and the “insect-associated” clade.

The first Jingmenvirus was first reported in 2014, with the discovery of Jingmen tick virus (JMTV) from Rhipicephalus microplus ticks collected in China (2). Since then, tick-associated Jingmenviruses close to the initial JMTV strain has been detected in numerous tick species (2, 7–11), in mosquitoes (2, 12), but also in vertebrates including cattle (MH133314.1) (13), monkey (14), rodents (9, 15), tortoise (ON158817.1) and humans with a history of tick bite (MN218697.1) (16). Serological tests have confirmed human exposure to JMTV in China (17). A low seroprevalence was also suggested in France (18). Other tick-associated Jingmenviruses, distant from the prototype JMTV strain identified in R. microplus, were discovered in mosquitoes, deer, bats, sheep, cattle, and in humans with febrile illness (6) which suggest that several viral species from the tick-associated clade are tick-borne arboviruses with zoonotic potential (18–21).

In contrast, Jingmenviruses from the “insect-associated” clade are generally considered as insect-specific viruses. These insect-associated Jingmenviruses have been detected in a broad range of invertebrates including mosquito, fly, flea, aphid, cricket, biting midge, and scorpion, but also in fungi and plants. The prototype strain of this clade, Guaico Culex Virus (GCXV), was isolated from pools of Culex mosquitoes collected in the Americas between 2008 and 2012 (14) but the virus was unable to replicate in vertebrate cell lines or in intracranially inoculated new-born mice, suggesting a restriction of the virus to its mosquito host (22).

Recently, the genome of a new Jingmenvirus strain belonging to the insect-associated clade was successfully assembled from the plasma of a 29-year-old HIV-1 and HBV-positive individual from Cameroon (Yaoundé region) (23). Despite the fact that Orf et al. could not demonstrate that the pathogenicity was due to this Jingmenvirus strain, the study was the first report of an “insect-associated” Jingmenvirus in vertebrates (23), suggesting that Jingmenviruses from the insect-associated clade could also infect vertebrates. The closest genomes of this new human-derived Jingmenvirus (HdJV) was the Shuangao insect virus 7 (SAIV7) isolated from a pool of flying insects from eastern China (24). The overall low identity between HdJV and SAIV7 (only 77% nucleotide identity of the conserved NSP1 coding for the viral polymerase) (5) indicated that HdJV constitutes a new species of Jingmenvirus. The initial discovery of HdJV reported a segmented genome constituted of four segments, but recent re-analysis of the sequencing data revealed the presence of a fifth segment, named segment 2–2, that appears to be much more conserved (93–99% nucleotide identity between HdJV and SAIV7) compared to other segments (5).

The vector of HdJV had not been identified. Herein, we report the detection and assembly of viral sequences with over 99% amino acid identity to HdJV in several mosquito species collected in Cameroon in 2022 and 2023, suggesting that mosquitoes may potentially constitute the missing vector host of HdJV.

## Methods

### a) Sampling plan and identification of mosquito species

Mosquitoes were collected in two different geographical areas in Cameroon: Garoua and Yaoundé. Yaoundé is the capital city of the country, located in the Centre region. It has a sub-equatorial Guinean climate with two distinct dry and rainy seasons, and with forest vegetation. Garoua is the capital of the North region. It has a tropical Sudanian climate with one rainy season which extends from May to November and predominantly savannah vegetation. Mosquito collections were conducted over a two-year period (2022–2023) during both the dry and rainy seasons.

Oral consent was obtained from the concession owners at each location. Mosquitoes were collected from around ten animal shelters (cattle, goats or sheep), using a Pokopack aspirator and/or a CDC light trap supplemented with CO_2_. Mosquito collections were also performed in livestock markets and abattoirs. The Prokopack aspirator was used to collect resting mosquitoes indoors and in surrounding vegetation. The CDC light trap was used to collect questing mosquitoes. Mosquitoes were anaesthetized by cooling and morphologically identified on an ice block using a magnifying glass. They were then pooled by species, season, and collection site with up to ten specimens per minipool. Monospecific pools were labelled and stored in liquid nitrogen in the field before being transferred at − 80°C in the laboratory until further experiments.

### b) Pooling and RNA Extraction

Minipools of mosquitoes were homogenized with 500 μL of PBS using a MagnaLyser version 1.1 (Roche, Mannheim, Germany) at 6,000 rpm for 1 min. Shreds were centrifuged for 2 min at 12,000× g and 4°C, then 167 μL of supernatant was transferred to 835 μL of RNA later solution (Invitrogen). The mixture was incubated overnight at 4°C and stored at − 80°C until shipment to Institut Pasteur in Paris. One hundred and forty-five minipools of female mosquitoes (representing a total of 1,075 female mosquitoes) were combined into 43 large pools according to the mosquito species, the season and the collection site to a maximum of 80 mosquitoes per large pool. Total RNA was extracted from the 43 large pools of mosquitoes in a Biosafety Level 3 (BSL-3) laboratory using the Maxwell RSC simply RNA tissue kit (Promega, Madison, WI, USA), according to the manufacturer’s instructions. RNA extracts were quantified with the Qubit RNA High sensitivity assay (Invitrogen, Waltham, MA, USA) and analyzed using an Agilent BioAnalyzer RNA pico chip (Agilent, Waldbronn, Germany).

Large pools were labelled according to the location (“Y” for Yaoundé and “G” for Garoua), species (“Aa”, “Ag”, “Cq” and “Cw” for *Aedes albopictus, Anopheles gambiae s.l., Culex quinquefasciatus*, and *Culex wansoni* respectively), year of sampling (“22” and “23” for 2022 and 2023), and season (“D” and “R” for the dry and rainy season respectively). Replicates were labelled “.1” and “.2” if more than one large pool with the same location, species, year and seasonal characteristics were sequenced.

### c) NGS Library Preparation and Sequencing

Sequencing libraries were prepared using the SMARTer Stranded Total RNA-seq kit v3-Pico input mammalian kit (Takara Bio, San Jose, CA, USA). The quantity of RNA input, the duration of heat fragmentation, and the final amplification were adapted according to each sample RNA profile. Quantification and quality controls of the libraries were verified by the Qubit DNA High sensitivity assay (Invitrogen) and the Bioanalyzer DNA High Sensitivity chips (Agilent, Waldbronn, Germany), respectively. Sequencing was carried out on the Illumina NextSeq 2000 devices in a paired-ends 2 × 100 bp format, to achieve approximately 50 million reads for each library.

### d) Viral assignment

Raw reads were processed with an in-house bioinformatics pipeline (Microseek (25)) that allows for quality check, read trimming, *de novo* assembly, and uses a series of BLAST-based similarity search, primarily against a curated protein reference viral database (RVDB-prot) for sensitive viral sequences detection (25, 26). This virome analysis revealed the presence of contigs with high identity to Jingmenvirus sp. strain Cameroon/U172471/201 (Human-derived Jingmenvirus, HdJV) in several samples. Sequenced reads were mapped against HdJV reference genome sequences (OQ835732, Seg1; OQ835733, Seg2; OQ835734, Seg3; OQ835735, Seg4; BK070268, Seg2-2) using Bowtie2 (27) and QIAGEN CLC Workbench (Version 23) in order to extract a consensus sequence per sample.

### e) Alignment and Phylogenetic analysis

Consensus sequences from each positive pool were manually verified using QIAGEN CLC Workbench (Version 23) before being aligned with other consensus sequences and 4 closely related viruses to verify the accuracy of each consensus. Because of the high nucleotide identity of the sequences generated from individual samples, a consensus genome was produced from all positive samples. The predicted protein NS5/NS5-like sequence was aligned using MAFFT (28) to all NS5/NS5-like protein sequences of all known Jingmenvirus species (23 species) and some closely related species (1). The alignment obtained was trimmed using trimAl, a tool for automated alignment trimming (Version 1.4.1) (29) for follow-up phylogenetic analyses.

All phylogenetic trees were built using PhyML with Smart Model Selection (Version 1.8.1) (30). The phylogenetic trees were constructed using the GTR + G model of nucleotides substitution. Tree topology was evaluated by the bootstrap method (1000 replicates). Trees were edited with iTol (Version 7.2) and were midpoint rooted when no outgroup was identified.

### f) Statistical analyses

Statistical analyses were performed using R software (R version 4.4.2) within the RStudio environment (version 2025.0.5). Descriptive statistical analysis was performed for mosquito species, regions, seasons, and collection years. Categorical data were summarized as percentages. Associations between the presence of Jingmenvirus and the different variables were assessed using the χ^2^ test or Fisher’s exact test. Statistical significance was defined as p < 0.05. The pooled prevalence for variable pool size and perfect tests was calculated using Epitools (31). This method estimates prevalence and confidence limits for variable pool sizes and assumes 100% test sensitivity and specificity (32).

## Results

As part of a metagenomics analysis aiming at deciphering the virome composition of mosquitoes from Cameroon, we identified eight contigs with length ranging from 313 to 1,359 nucleotides that had predicted protein homology with Jingmenvirus sp. strain Cameroon/U172471/201 (Human-derived Jingmenvirus, HdJV) and amino acid identity ranging from 98.6–100%.

HdJV-related reads were detected in abundance in 6 pools of mosquitoes, with sequences covering at least three genome segments (Table 1). These samples corresponded to pools of *Culex quinquefasciatus*, *Aedes albopictus* and *Culex wansoni* collected in Yaoundé during the two seasons (rainy and dry) of the two years of collect (2022 and 2023) (Table 1). Traces (no more than two reads) of HdJV were detected in two more samples, including a pool of *Anopheles gambiae s.l*. from Garoua (Table 1). No Jingmenvirus sequence was detected in the following mosquito species: *Aedes aegypti, Aedes vittatus, Culex duttoni, Culex rubinotus, Culex tritaeniarynchus and Mansonia africana*. However, among these additional mosquito species, only *Aedes aegypti* and *Culex tritaeniarynchus* were sampled in Yaoundé.

The overall pooled prevalence of mosquito-associated Jingmenvirus (MaJV) was estimated to 0.90% [0.41–1.69] (Table 2). In *Culex quinquefasciatus* that was sampled more often than any other mosquito species (N = 29 pools), the overall pooled prevalence was estimated to 0.59% [0.21–1.28] but reached 1.69% [0.59–3.82] when considering only mosquitoes sampled in Yaoundé (5/8 pools positive, Table 2). The unique variable significantly associated with MaJV detection was the sampling area, Yaoundé showing the highest prevalence (2.29% [0.95–4.68]) compared to Garoua (0.18% [0.01–0.79]) (p-value = 0.001, Table 2).

**Figure 1:**
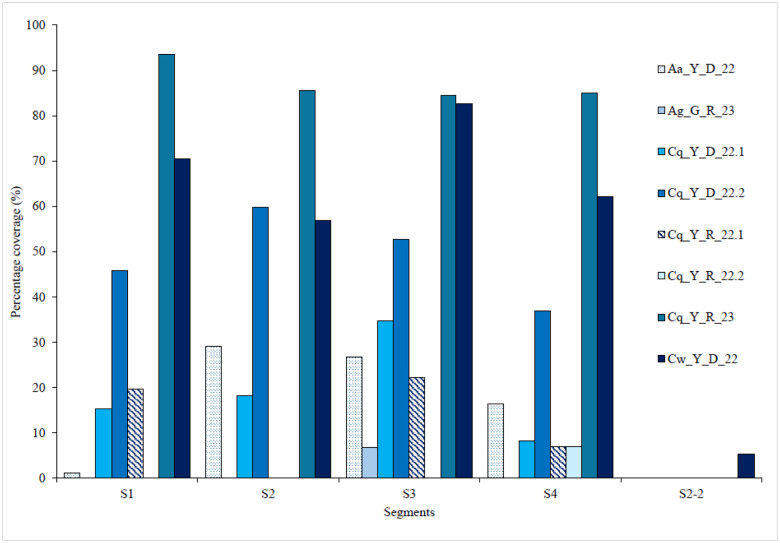
Percentage coverage of the reference sequences for the eight positive pools (Human-derived Jingmenvirus OQ835732- OQ835735 and BK070268)

**Figure 2:**
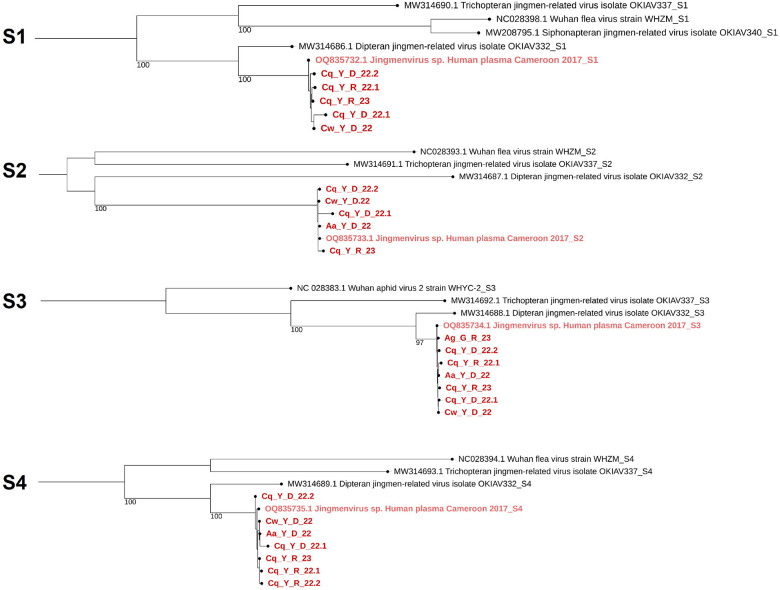
Phylogenetic trees of the different positive pools’ consensus using a maximum likelihood analysis for the segment 1 to 4 Jingmenviruses nucleotide sequences (PhyML 3.0). The phylogenetic tree was built using the Model of nucleotides substitution: GTR, gamma distributed with bootstraps (branch labels) and midpoint rooted using iTol (Version 7.2).

The genome coverage of each segment of MaJV is presented in Fig. 1 for all eight positive pools. Samples from *Culex wansoni* (Cw_Y_D_22) and *Culex quinquefasciatus* (Cq_Y_D_22.2) showed the highest genome coverage. Of note, traces of segment 2–2 were only detected in *C. wansoni*. Consensus sequences generated from individual samples showed 98.8 to 100% nucleotide identity (Supplemental Fig. 1). Phylogenetic analyses performed at the nucleotide level confirmed that viral sequences originating from different samples belonged to the same clade (Fig. 2).

Knowing that the eight strains of MaJV sequences represent the same viral species, reads belonging to each segment were combined to assemble a single consensus genome of MaJV (Supplemental Fig. 2; Genbank: PV953369, PV953370, PV953371, PV953372). MaJV genome segments presented with 96.4 to 98.9% nucleotide identity to HdJV (Supplemental Fig. 1). The coverage percentage for each segment ranged from 93.4–98.1%.

**Figure 3:**
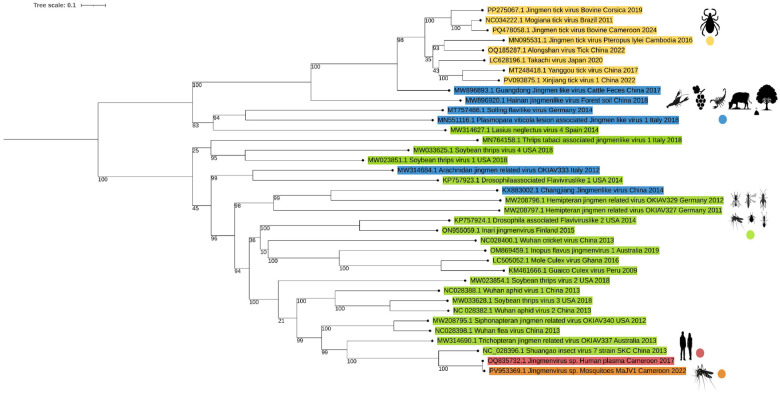
Maximum likelihood analysis of segment 1 Jingmenviruses amino acid sequences of RDRP/NS5/NS5-like gene was realized using PhyML 3.0. The phylogenetic tree was constructed using the Model of nucleotides substitution: GTR, gamma distributed with bootstraps (branch labels) and midpoint rooted using iTol (Version 7.2). Sequences are color-coded according to their origin: tick-associated viruses are shown in yellow, insect-associated viruses in green, and sequences from environmental or unclassified sources in blue. Two sequences of particular interest are highlighted one in orange, corresponding to the Jingmenvirus strain detected from a mosquito in Cameroon (MaJV of this study PV953369.1), and one in pink, representing a previously reported Jingmenvirus sequence from a human plasma sample collected in Cameroon in 2017 (GenBank accession OQ835732.1).

Phylogenetic analysis of Jingmenviruses’ viral polymerase confirmed that MaJV (PV953369.1) clusters very closely to HdJV (OQ835732.1) and is distinct from other insect-associated Jingmenviruses (Fig. 3). At the root of the clade formed by MaJV and HdJV is placed Shuangao insect virus 7 (SAIV7). Supported by a high bootstrap value, viruses of the same clade were detected in cat fleas from USA, China and in caddisflies from Australia distant from tick-associated Jingmenviruses.

## Discussion

The present study represents the first detection of a Jingmenvirus in mosquitoes from Cameroon, significantly expanding the known geographical distribution and host range of this viral group. Phylogenetic analyses of the viral polymerase revealed that the Mosquito-associated Jingmenvirus (MaJV) sequences fall within the “insect-associated” Jingmenvirus clade. Most strikingly, MaJV presents very high nucleotide identity to the HdJV initially discovered within human plasma sample from Yaoundé (33). With 96.4 to 98.9% nucleotide identity depending on the segments, the two viruses could be considered as different strains of the same species. These results strongly suggest the potential transmission of HdJV from mosquitoes to humans. Considering that MaJV (and its human counterpart) belong to the insect-associated Jingmenvirus clade, that clade may no longer be restricted to insects. The detection of MaJV in mosquito species known for their vectorial capacity (34–37) underscores the importance of evaluating the zoonotic transmission risk of HdJV and the potential role of mosquitoes as vectors of this novel arbovirus..

The ecological diversity of HdJV and its potential for widespread distribution within several mosquito species within the region further support its zoonotic potential. Interestingly, the MaJV strain was detected in diverse mosquito species (i.e. *Aedes albopictus, Anopheles gambiae s.l., Culex quinquefasciatus* and *Culex wansoni*) suggesting that these mosquitoes could have been infected during blood feeding onto a common viremic vertebrate host. Indeed, *Aedes albopictus* feeds predominantly on mammalian hosts, including humans (upper to 80%), cats, dogs and more rarely birds (38–40). *Culex quinquefasciatus*, like the other species of *Culex* mosquitoes, is a typical ornithophilic mosquito, but opportunistically bites dogs, humans, and sometimes other mammals (41–43). *Anopheles gambiae s.l*. is considered as the world’s most important malaria vector and is well-established as highly anthropophilic (44–46). Similarly, vector competence studies should be conducted to assess the infection rate, dissemination and transmission of this virus by different mosquito species and to determine if one mosquito species constitute the main vector of HdJV while the others might be accidentally infected during blood feeding onto a viremic host.

MaJV was mostly detected in Yaoundé, during both the dry and the rainy seasons in 2022 and 2023. The overall pooled prevalence of MaJV was estimated at 0.90% [0.41–1.69] in our study. The same high order of pooled prevalence (around 0 to 1%) were observed for other flaviviruses including in endemic regions for their circulation (47–49). The prevalence was significantly higher in Yaoundé (2.29% [0.95–4.68]) compared to Garoua (0.18% [0.01–0.79]) indicating geographical variation in virus prevalence. This is particularly evident for *Culex quinquefasciatus* species for which no positive pool was found among the 21 originating from Garoua whereas MaJV was detected in 5 of the 8 pools from Yaoundé. In our study, MaJV detection was not related to seasonality, which conflicts with the existing literature. Indeed, temperature is one of the most important environmental factor affecting biological processes of mosquitoes, including their interactions with viruses and susceptibility to pathogen infection (50, 51). Given the limited number of samples per mosquito species, the relatively small number of pools with MaJV, and that our screening focused only on two sites, these measures remain preliminary and further studies are needed to determine the extent of the host range of MaJV, its geographic distribution and the impact of seasonality on MaJV circulation.

Further research is now necessary to assess the zoonotic potential of MaJV through a combination of experimental infection studies and epidemiological surveillance. It should be noted that the detection of HdJV in plasma of an immunocompromised patient could reflect the low infectivity of this virus to humans. Moreover, numerous viruses have been shown to establish persistent infections and prolonged viral shedding in immunocompromised individuals (52–54). This phenomenon is well-characterized not only for SARS-CoV-2 but also for a wide range of other viral pathogens (55). The understanding of viral evolution and mutational dynamics within these hosts, as well as the potential global implications, is essential particularly considering the growing population of immunocompromised patients worldwide (56). Of note, serological investigations may be employed to assess human exposure within Cameroon, with a particular focus on Yaoundé where the virus prevalence appears to be higher. Monitoring the circulation of MaJV in vectors, potential animal hosts, and humans will be crucial for understanding the risk that this virus represents to public health and implementing evidence-based control measures.

## Supplementary Files

This is a list of supplementary files associated with this preprint. Click to download.

- SupplementFigureS1.pdf
- SupplementFigureS2.pdf
- SupplementaryTable1.docx

## Figures and Tables

**Table 1:** Description of positive pools for Jingmenvirus detection according to the different consensus by segment.

Sample code	Species Mosquitoes	Region	Season	Year	Number of mosquitoes by pool	Consensus length / Read count					Total Read count
						S1 2955 bp	S2 1,603 bp	S3 2,671 bp	S4 2,585 bp	S2–2 1,693 bp	
Aa_Y_D_22	*Aedes albopictus*	Yaounde	Dry	2022	17	33 / 1	467 / 8	712 / 15	424 / 14	ND	38
Ag_G_R_23	*Anopheles gambiae s.l*.	Garoua	Rainy	2023	10	ND	ND	180 / 2	ND	ND	2
Cq_Y_D_22.1	*Culex quinquefasciatus*	Yaounde	Dry	2022	69	451 / 14	293 / 12	930 / 22	213 / 3	ND	51
Cq_Y_D_22.2	*Culex quinquefasciatus*	Yaounde	Dry	2022	76	1,354 / 24	958 / 20	1,409 / 31	954 / 29	ND	104
Cq_Y_R_22.1	*Culex quinquefasciatus*	Yaounde	Rainy	2022	76	ND	ND	593 / 13	179 / 3	ND	23
Cq_Y_R_22.2	*Culex quinquefasciatus*	Yaounde	Rainy	2022	80	ND	ND	ND	181 / 2	ND	2
Cq_Y_R_23	*Culex quinquefasciatus*	Yaounde	Rainy	2023	22	2,765 / 238	1,371 / 56	2,256 / 180	2,199 / 177	ND	651
Cw_Y_D_22	*Culex wansoni*	Yaounde	Dry	2022	2	2,084 / 47	911 / 28	2,206 / 90	1,607 / 56	91 / 2	223

ND: not detected

**Table 2:** Positivity rate of pools for MaJV detection according to the different variables, univariate analysis and pooled prevalence of MaJV.

Variables		MaJV detection			p-value	Pooled prevalence of MaJV
		n	N	%		
Species	*Aedes aegypti*	0	4	0	0.43	0
	*Aedes albopictus*	1	2	50.0		10.56 [0.56–55.31]
	*Aedes vittatus*	0	1	0		0
	*Anopheles gambiae s.l*.	1	2	50.0		21.32 [1.11–89.5]
	*Culex duttoni*	0	1	0		0
	*Culex quinquefasciatus*	5	29	17.2		0.59 [0.21–1.28]
	*Culex quinquefasciatus (from Yaoundé)*	5	8	62.5		1.69 [0.59–3.82]
	*Culex rubinotus*	0	1	0		0
	*Culex tritaeniarynchus*	0	1	0		0
	*Culex wansoni*	1	1	100		/
	*Mansonia africana*	0	1	0		0
Season	Dry	4	21	19.0	0.99	0.85 [0.26–1.99]
	Rainy	4	22	18.2		0.96 [0.30–2.25]
Areas	Yaounde	7	15	46.7	0.001	2.29 [0.95–4.68]
	Garoua	1	28	3.6		0.18 [0.01–0.79]
Year	2022	6	16	37.5	0.057	1.24 [0.49–2.55]
	2023	2	27	7.4		0.76 [0.19–1.97]
Overall		8	43	18.6	/	0.90 [0.41–1.69]

n = number of positive pools; N = overall number of pools by variables; % = the detection rate

## Data Availability

Sequences of the four segment of the virus Genbank accession number: PV953369, PV953370, PV953371, PV953372.
